# Assessment of the Optic Nerve Head Parameters Using Heidelberg Retinal Tomography III in Preterm Children

**DOI:** 10.1371/journal.pone.0088056

**Published:** 2014-02-13

**Authors:** Salem Alshaarawi, Ismail Shatriah, Embong Zunaina, Wan Hazabbah Wan Hitam

**Affiliations:** Department of Ophthalmology, School of Medical Sciences, Universiti Sains Malaysia, Kubang Kerian, Kelantan, Malaysia; Faculty of Medicine University of Leipzig, Germany

## Abstract

**Background:**

Variations in optic nerve head morphology and abnormal retinal vascular pattern have been described in preterm children using digital image analysis of fundus photograph, optical coherence tomograph and serial funduscopy. We aimed to compare the optic nerve head parameters in preterm and term Malay children using Heidelberg Retinal Tomograph III.

**Design:**

A cross sectional study.

**Methodology/Principal Findings:**

Thirty-two preterm Malay children who were born at up to 32 weeks postconception, and 32 term Malay children aged 8–16 years old were recruited into this cross sectional study, which was conducted in the Hospital Universiti Sains Malaysia, Malaysia from January to December 2011. Their optic nerves were scanned and analyzed using a Heidelberg Retinal Tomography (HRT) III (Heidelberg Engineering, Germany). Preterm children showed an increased rim volume (SD) (0.56 (0.26) vs 0.44 (0.18) mm^3^, respectively), smaller cup shape (SD) (0.18 (0.07) vs 0.25 (0.06) mm, respectively), increased height variation contour (SD) (0.44 (0.14) vs 0.35 (0.08) mm, respectively), and increased cup depth (SD) (0.24 (0.11) vs 0.17 (0.05) mm^3^, respectively) when compared to their normal peers (p<0.05). There were no significant differences in the mean disc area, cup area, cup to disc ratio or rim area between the preterm and term children (p>0.05) in our study.

**Conclusions/Significance:**

Preterm children exhibit different characteristics of optic nerve head parameters with HRT III analysis. Increased cup depth in preterm children suggests a need for close observation and monitoring. It may raise suspicion of pediatric glaucoma when proper documentation of intraocular pressure and clinical funduscopy are unsuccessful in uncooperative children.

## Introduction

The use of newer technologies such as digital analysis of fundus photographs, retinal and optic nerve head imaging, and RetCam image analysis have improved the diagnostic accuracy and management of pediatric eye diseases [1]–[12]. Variations in optic nerve head morphology and abnormal retinal vascular pattern have been described in preterm children using the above modalities [4]–[5], [7]–[12].

However, the above studies were confined mainly to preterm infants and/or children using digital image analysis of fundus photograph, optical coherence tomography (OCT) and serial funduscopy [4]–[5], [7]–[13]. Based on a PubMed search, no published studies have performed optic nerve analysis using Heidelberg Retinal Tomography (HRT) of preterm children. The present study aimed to compare the optic nerve head characteristics of preterm and term children in the suburban Kota Bharu of Peninsular Malaysia.

## Materials and Methods

This was a cross-sectional study involving 64 Malay children aged 8 to 16 years old; 32 preterm and 32 term children. This study was conducted in the Hospital Universiti Sains Malaysia from January, 1^st^ until December, 31^st^ 2011, according to the Declaration of Helsinki. The study protocol was approved by the Research and Ethics Committee, School of Medical Sciences, Universiti Sains Malaysia and conformed to the provisions of the Declaration of Helsinki.

A list of babies born from 1999 to 2003 was obtained from Labor Ward, Neonatal Ward and Record Office of the Hospital Universiti Sains Malaysia, Malaysia. The parents were contacted via telephone, and their children were invited to participate in our study. The non-probability sampling method was applied. The children were selected based on their ease of access to the hospital and subsequent clinical examination. Written informed consent was obtained from all parents in both groups before participation of their child.

The children were included into the preterm group if they were born at 32 weeks postconception or less, were of Malay ethnicity for at least 2 generations (i.e. all of the parents and grandparents were Malay) and displayed normal anterior and posterior segment findings based on a thorough eye examination. Malay children who were born at 38 weeks of gestation or more and had normal anterior and posterior segment findings were allocated to the control group (term children).

After initial recruitment, 32 children were included in each group for analysis after exclusion of possibility of optic nerve abnormalities, refractive error based on spherical equivalent of ±4.0 diopter, evidence of compressive or hereditary optic neuropathy, trauma, infiltrative disease, congenital glaucoma, retinitis pigmentosa, systemic illnesses or developmental delay. Previous documentation of retinopathy of prematurity (ROP) were excluded from the preterm group.

There were 43 preterm children in the initial list. 36 preterm children attended our clinic, while the remaining were either refused, non-contactable based on their previous contact numbers, having difficulty with transportation to hospital or moving to other districts. 4 children were excluded due to previous corneal injury, anisometropia and high myopia. 32 term children were allocated to the control group. They were age and gender matched. The similar exclusion criteria were applied to the control group.

All the children were tested for their distant and near visual acuity. A cover test was performed to detect the presence of tropia. They were checked for afferent pupillary defect, and both the anterior and posterior segments were assessed carefully to rule out any apparent ocular pathology. The intraocular pressure was measured in both eyes. Cycloplegic refraction was performed in all children prior to the imaging study.

The optic nerve head parameters were assessed using a HRT III (Heidelberg Engineering, Germany). All preterm and term children were scanned by an identified masked examiner using the eye-tracking feature. The right eye was chosen for analysis in both groups of patients. The parameters included the disc area, cup area, rim area, cup/disc area ratio, linear cup/disc area ratio, mean cup depth, maximum cup depth, height variation contour, cup shape measurement, and contour line.

We captured the optic nerve head images from a non-dilated pupil. The image was considered of good quality according to the criteria described by Bowd et al. [14]. These included the optic disc image appearing centrally, minimal eye movement detected during image capture, the absence of artefacts and a topography standard deviation less than 50 micrometres [14].

The demographic data, clinical findings and optic nerve head parameters were documented in a separate data collection sheet. The data were analyzed using the Statistical Package for Social Sciences version 18.0. The mean and standard deviation (SD) were described for a normally distributed data, while the median and interquartile range (IQR) were reported for a skewed data. Categorical variables were described in terms of frequency and percentages. Independent t-tests were used to compare the mean of optic nerve head parameters between preterm and term Malay children. A Mann-Whitney test was used to compare the optic nerve head parameters according to the age and gender. Data on intraocular pressure will be published in a subsequent manuscript.

## Results

Table 1 shows the gestational age, birth weight and presence of previous ROP in the preterm children. The majority of them were born between 30 to 32 weeks of gestation and had a birth weight ranging from 1000 to 1500 gram. The mean age (SD) were 9.8 (2.60) years for preterm children respectively and 11.0 (2.41) years for the term children. Visual acuity of 6/12 (20/40) or worse were noted in 9.3% of the preterm only. Three preterm children were found myopic and their spherical equivalent ranged between −2.0 to −2.5 diopter. None of the term children had visual acuity worse than 6/9 (20/30). These are summarized in Table 2.

**Table 1:** Demographic data of the preterm children.

		Preterm Children	
		Male	Female
		n (%)	n (%)
Gestational Age (week)	24–26	3 (9.4)	1 (3.1)
	27–29	3 (9.4)	2 (6.2)
	30–32	12 (37.5)	11 (34.4)
Birth Weight (gram)	501–1000	6 (18.7)	3 (9.4)
	1001–1500	12 (37.5)	11 (34.4)
Presence of ROP	Yes	0 (0.0)	0 (0.0)
	No	18 (56.2)	14 (43.7)

**Table 2:** Distribution of the age, gender and visual acuity.

		Preterm		Term	
		Male	Female	Male	Female
Age (years) (n, %)	8–10	11 (34.3)	9 (28.1)	12 (37.5)	6 (18.7)
	11–13	6 (18.7)	3 (9.3)	2 (6.3)	6 (18.7)
	14–16	1 (3.1)	2 (6.2)	4 (12.5)	2 (6.2)
Mean (years) (SD)*		9.8 (2.60)		11.0 (2.41)	
Visual acuity (n, %)	6/6–6/9	16 (50.0)	13 (40.7)	18 (56.2)	14 (43.8)
	6/12–6/15	2 (6.2)	1 (3.1)	0 (0.0)	0 (0.0)
	Worse than 6/15	0 (0.0)	0 (0.0)	0 (0.0)	0 (0.0)

SD, standard deviation.

Table 3 shows a comparison of optic nerve head parameters between the preterm and term groups. There were statistically significant differences of rim volume (p = 0.01), cup shape measurement (p<0.001), height variation contour (p = 0.01), and mean cup depth (p = 0.01) between the two studied groups. However, no significant differences were observed in the mean disc area, cup area, rim area, cup volume, cup to disc ratio or maximum cup depth (p>0.05). We further analyzed the optic nerve head parameters of the preterm and term children according to the age and gender, and we found no statistically significant differences (p>0.05).

**Table 3:** Comparison of optic nerve head parameters between the preterm and term children.

Variables		Mean (SD)	Mean diff (95% CI)	t-stat (df)	*p-value
Disc area (mm^2^)	Preterm	2.70 (0.73)			
	Term	2.44 (0.47)	0.27 (−0.58, 0.04)	1.75 (53.1)	0.08
Cup area (mm^2^)	Preterm	0.67 (0.42)			
	Term	0.55 (0.29)	−0.13 (−0.13, 0.05)	−1.38 (62.0)	0.17
Rim area (mm^2^)	Preterm	2.03 (0.67)			
	Term	1.89 (0.46)	−0.15 (−0.44, 0.15)	−0.97 (62.0)	0.33
Cup volume (mm^3^)	Preterm	0.18 (0.22)			
	Term	0.11 (0.09)	−0.06 (−0.15, 0.02)	−1.59 (62.0)	0.12
Rim volume (mm^3^)	Preterm	0.57 (0.26)			
	Term	0.44 (0.18)	−0.14 (−0.25, −0.03)	−2.59 (54.9)	**0.01**
Cup/Disc area	Preterm	0.24 (0.11)			
	Term	0.22 (0.11)	−0.03 (−0.08, 0.04)	−0.81 (62.0)	0.41
Linear Cup/Disc	Preterm	0.48 (0.13)			
	Term	0.45 (0.12)	−0.03 (−0.09, 0.04)	−0.78 (62.0)	0.43
Cup shape measurement (mm^3^)	Preterm	0.18 (0.07)			
	Term	0.25 (0.06)	−0.07 (−0.09, −0.04)	−3.83 (62.0)	**<0.001**
Height variation countour (mm^3^)	Preterm	0.44 (0.14)			
	Term	0.35 (0.08)	−0.09 (−0.15, −0.03)	−2.76 (62.0)	**0.01**
Maximum cup depth (mm^3^)	Preterm	0.61 (0.20)			
	Term	0.56 (0.19)	−0.05 (−0.16, 0.05)	−1.06 (62.0)	0.29
Mean cup depth (mm^3^)	Preterm	0.24 (0.11)			
	Term	0.17 (0.05)	−0.07 (−0.11, −0.02)	−2.75 (45.1)	**0.01**

* Independent t-test<0.05 was considered statistically significant.

## Discussion

In this hospital-based sample, we present new data of optic nerve head parameters using HRT III of preterm children aged 8–16 years of Malay ethnicity. Previous published studies of preterm children had variable inclusion criteria and used different instruments to assess the optic nerve parameters [4]–[13]. Thus, a parallel comparison is relatively difficult and of debatable utility [15]–[17]. Table 4 compares the optic nerve head parameters of our subjects with the other published data for subjects with a relatively similar range of current age, gestational age and birth weight [5], [7]–[9]. To our knowledge, there is no published study analyzing optic nerve head parameters using HRT III in preterm children.

**Table 4:** Comparison of the mean values of the optic nerve head parameters in the preterm children with other published studies.

Parameters		Current study (Malaysia, 2014)	Wirkstrand et al (Sweden, 2010)^7^	Hellström et al (Sweden, 2000)^8^	Hellström et al (Sweden, 1997)^9^	Samarawickrama et al (Australia, 2009)^5^
Number of participants		32 subjects	53 subjects	50 eyes	39 eyes	71 eyes
Birth wight (gram)		500 to1499	1063	450 to1520	600 to 2200	<2500
Current age	Mean (SD)	9.81 (2.60)	5.4 years**	4.8 years	4.8 years	12.7 (12.58–12.79)*
	Range	8–16 years	4.8–6.1 years	5.1–9.3 years	3.1–9.1 years	12 years
Born at gestational age (weeks)		<32	27.1**	27	24–32 weeks	<37
Instrument used		HRT III	Digital image analysis of fundus photograph	Digital image analysis of fundus photograph	Digital image analysis of fundus photograph	OCT
Mean optic disc area (SD), mm^2^		2.70 (0.73)	2.2 (0.3)	2.35**	2.80 (0.46)**	2.32 (2.34–2.41)*
Mean cup area (SD), mm^2^		0.67 (0.42)	0.4 (0.3)	0.36**	0.34 (0.27)	0.45 (0.39–0.52)*
Mean cup/disc ratio (SD)		0.24 (0.11)	NA	NA	NA	0.20 (0.17–0.22)*
Mean rim area (SD), mm^2^		2.03 (0.67)	1.7 (0.4)	2.03	NA	NA
Mean cup volume (SD), mm^3^		0.18 (0.22)	NA	NA	NA	NA
Mean rim volume (SD), mm^3^		0.57 (0.26)	NA	NA	NA	NA
Mean cup depth (SD), mm^3^		0.24 (0.11)	NA	NA	NA	NA
Mean maximum cup depth (SD), mm^3^		0.61 (0.20)	NA	NA	NA	NA
Mean cup shape measurements (SD), mm		−0.18 (0.07)	NA	NA	NA	NA
Mean height variation contour (SD), mm		0.44 (0.14)	NA	NA	NA	NA
Mean linear cup disc (SD), mm		0.48 (0.13)	NA	NA	NA	NA

SD, standard deviation; HRT, Heidelberg Retinal Tomography; Optical Coherent Tomography; NA, data not available.

* Means and 95% confidence interval.

** Median.

We found no significant difference of the mean optic disc areas of the two groups (p value>0.05) using the HRT III analysis. Our finding is consistent with an observation by Samarawickrama et al. who reported that optic disc area displayed no significant differences between low birth weight and normal birth weight children (p = 0.64) using Stratus OCT software [5].

The earlier published studies on optic disc morphology in preterm children were performed using digital analysis of fundus photographs [7]–[9]. Hellström et al. in 1997 reported similar observation as ours, the median optic disc areas of 2.80 mm^2^ in the preterm children and 2.87 mm^2^ in the control group ranging from 3.1 to 9.1 years of age (p-value insignificant) [9]. They compared the fundus photograph of 39 Swedish preterm children who were born 29 weeks of gestation to term children.

In contrast, the other two studies demonstrated that preterm children had a smaller optic disc area than term children [7], [8]. Wirkstrand et al. examined 53 preterm Swedish children aged 5.4 years old who were born at less than 32 weeks of gestation [7], while Hellström et al. evaluated 45 fundus photographs of preterm children aged 7 years old who were born between 24–28 weeks of gestation [8]. The latter two studies had included preterm children with ischemic brain injury [7], [8]. Jacobson et al. estimated that those preterm children who have had a brain lesion before 28 weeks of gestation had a smaller optic disc area [18].

The mean optic disc area has also been assessed in preterm infants by several researchers using other diagnostic tools [6], [10]–[11]. Rimmer et al. conducted a post mortem examination of 20 eyes of preterm infants who were born at less than 40 weeks of gestation and reported that the mean optic disc area was 0.82 (0.26) mm^2^ [6]. De Silva et al. performed digital retinal imaging in preterm infants and reported that the mean optic disc areas was 1.17 (0.26) mm^2^ in preterm infants born at 30 weeks of gestation [10]. McLoone et al. reported that the mean optic disc area was 1.13 mm^2^ in the infants born at 28 weeks of gestation using RetCam image analysis [11]. However, the above studies were confined to preterm infants only.

We observed no significant difference between the mean cup area in the preterm and the term children (p>0.05). Our finding is consistent with observation by Hellström et al. [9]. However, our result contradicts previously published studies, as they observed that the preterm children demonstrated larger cups size [5], [7]–[8], [13], [19]. Wikstrand et al. noted the cup area was significantly larger in the preterm children than in term children (the mean cup size was 0.4 (0.3) mm^2^ vs 0.3 (0.2) mm^2^, p = 0.0027) [7]. Both Park et al. and Fledelius also noted increased cupping in low birth weight and premature children [13], [19].

Both the preterm and term children in our study have displayed comparable cup to disc ratios, 0.24 (0.11) and 0.22 (0.11), respectively (p>0.05). This finding suggests that both the optic disc and cup areas in our studied population were consistently large. Our observation is parallel with a report by Huynh et al. They observed that the disc and cup dimensions were significantly larger in East Asian children aged 6 year old when compared to European white and Middle Eastern children [20]. The study by Samarawickrama et al. is the only published study that described the cup to disc ratio in preterm children [5]. The other published studies did not address this parameter [7]–[9].

Samarawickrama et al. reported a contradictory finding compared to ours, they noted that the cup to disc ratio was significantly larger in low birth weight children (0.20, ranged 0.17–0.22) than in normal birth weight children (0.17, ranged 0.16–0.18), (p = 0.04) [5]. Our inclusion criteria were slightly different from their study. They assessed the preterm children who were born at less than 37 weeks and had birth weight of less than 2500 grams using the OCT machine. Of the 90 children with low birth weight who were recruited into their study, there were 20 East and South Asian children, and the remaining were Caucasian and Middle Eastern children. We postulate that the above variables may contribute to the differences between their findings and ours.

A progressive cupping in children is probably not a worrying sign in children as reported by Park et al. [13]. They compared the optic cup progression of preterm and term children in serial fundoscopic examination in a longitudinal study of a minimum 5 years period of observation. They observed that a non-significant trend of progressive cupping of the two studied groups and concluded that progression of cup to disc ratio was not a specific sign of glaucoma in children [13]. We believe that this is another issue that needs further evaluation in the preterm children. The use of newer technology and software is expected to contribute to new knowledge that brings impact to the clinical practice.

We observed that the preterm children born at 32 weeks and less have increased rim volume, height variation contour and cup depth but smaller cup shape when compared to their normal peers (p<0.05). These observations have not been reported before. It is relatively difficult for us to be certain of their clinical importance at present. Perhaps there will be more published studies of this topic in future that will provide us with clear details of their significance in clinical practice and management.

The strengths of our study are that it provides new information and detailed analysis on the optic nerve of preterm children using HRT III analysis. We found that the HRT III measurements were quick, easy and tolerable by our studied children. This observation was also consistent with that of Larsson et al., who examined normal children with an age range fairly similar to that in our study [21].

In conclusion, preterm children have an increased rim volume, height variation contour and cup depth, but the cup shape was observed smaller. The disc area, cup area and cup to disc ratio were similar in both preterm and term children. An increased cup depth in preterm children merits attention and suggests a need for long term monitoring. It may raise a suspicion of pediatric glaucoma and leads to overdiagnose the condition especially when failing to document proper intraocular pressure measurement and optic disc examination in such uncooperative children.
